# Telemedicine Improves the Short-Term Medical Care of Acute ST-Segment Elevation Myocardial Infarction After Primary Percutaneous Coronary Intervention

**DOI:** 10.3389/fcvm.2021.693731

**Published:** 2021-07-12

**Authors:** Heba Kamel, Mohamed Saber Hafez, Islam Bastawy

**Affiliations:** Department of Cardiology, Faculty of Medicine, Ain Shams University, Cairo, Egypt

**Keywords:** drug adherence, major adverse cardiac events, telecardiology, digital health, acute coronary syndrome, videoconferencing teleconsultations

## Abstract

**Objectives:** Telemedicine appears to be a promising tool for healthcare professionals to deliver remote care to patients with cardiovascular diseases especially during the COVID-19 pandemic. We aimed in this study to evaluate the value of telemedicine added to the short-term medical care of acute ST-segment elevation myocardial infarction (STEMI) after primary percutaneous coronary intervention (PCI).

**Methods:** Two hundred acute STEMI patients after primary PCI were randomly divided into two groups. One hundred patients in group A (study group) received a monthly videoconferencing teleconsultation using a smartphone application for 3 months starting 1 week after discharge and at least a single face-to-face (F2F) clinic visit. We reviewed in each virtual visit the symptoms of patients, adherence to healthy lifestyle measures, medications, smoking cessation, and cardiac rehabilitation. Group B (control group) included 100 patients who received at least a single F2F clinic visit in the first 3 months after discharge. Both groups were interviewed after 4 months from discharge for major adverse cardiac events (MACE), adherence to medications, smoking cessation, and cardiac rehabilitation. A survey was done to measure the satisfaction of patients with telemedicine.

**Results:** There was no significant difference between both groups in MACE and their adherence to aspirin, P2Y12 inhibitor, and beta-blockers. However, group A patients had better adherence to statins, angiotensin-converting enzyme inhibitors or angiotensin receptor blockers, smoking cessation, and cardiac rehabilitation. Sixty-one percent of patients stated that these videoconferencing teleconsultations were as good as the clinic visits, while 87% of patients were satisfied with telemedicine.

**Conclusions:** Telemedicine may provide additional benefit to the short-term regular care after primary PCI to STEMI patients through videoconferencing teleconsultations by increasing their adherence to medications and healthy lifestyle measures without a significant difference in the short-term MACE. These virtual visits gained a high level of satisfaction among the patients.

## Introduction

Acute myocardial infarction (AMI) is considered one of the leading causes of morbidity and mortality worldwide. Major adverse cardiovascular events (MACE) after AMI have no certain exact definition, but over time numerous definitions have been used in cardiovascular research, with MACE used as primary or secondary endpoint. Several adverse events included in different research as a component of MACE are heart failure (HF), non-fatal re-infarction, recurrent anginal pain, re-hospitalization for cardiovascular-related illness, repeat percutaneous coronary intervention (PCI), coronary artery bypass grafting (CABG), and all-cause mortality. MACE can also include unscheduled coronary revascularization, stroke, re-infarction, and all-cause death and mortality (1).

After discharge, patients with myocardial infarction (MI) have a life-long journey with lifestyle measures, coronary artery disease (CAD) risk factor control, and pharmacological treatment to reduce the risk of morbidity and mortality. All patients should be involved in an exercise-based cardiac rehabilitation program that is associated with better outcomes (2). On the contrary, low adherence to treatment in these patients is associated with worse outcomes. Low adherence to treatment is challenging, and continuous effort is required from healthcare professionals to maintain adequate communication with patients to enforce the importance of good adherence to medications and healthy lifestyle measures (3).

Telemedicine is a tool that uses information and communication technology to provide remote healthcare services (4). It has the potential to improve patient healthcare outcomes, provide access to medical services, and reduce healthcare costs. The application of telemedicine has been facilitated by continuous advances in technology and the increased spread of Internet services. As telemedicine application continues to evolve, it is important to understand the impact that telemedicine might have on patients, healthcare professionals, and the organization of care (5). Cardiology is considered to be one of the first branches in medicine where telemedicine systems have been applied (6). Telemedicine has a potentially broad application to cardiovascular disease (CVD), and it might have an important role as part of a strategy for the delivery of effective healthcare for patients with CVD, especially those with MI that is considered a leading cause of mortality and morbidity (7). Many studies showed that telemedicine is widely used in the cardiovascular field, such as in remote blood pressure (BP) monitoring (8), pre-hospital electrocardiography in the management of MI (9), monitoring of arrhythmias (7), monitoring of anticoagulant therapy (10), and regular follow-up of chronic cardiac conditions such as in heart failure and following MI or PCI, especially following the coronavirus disease 19 (COVID-19) pandemic (11, 12).

Despite the ease of receiving telemedicine services and the reduced cost of travel to hospitals and clinics, F2F contact between clinicians and patients is somewhat preferred, but with the emergence of the COVID-19 pandemic recently and the increased need for social distancing, telemedicine may present an acceptable alternative to F2F contact in non-emergent medical situations and regular follow-ups in addition to a possible beneficial add-on effect to traditional healthcare services. In this study, we aimed to evaluate the role of telemedicine in the short-term medical follow-up after the discharge of patients who underwent primary PCI for AMI by providing regular virtual visits, followed by collecting data from the patients.

## Materials and Methods

This was a randomized controlled study that initially included 273 patients who presented to Ain Shams University Hospital with acute STEMI and underwent primary PCI starting from June 2020 till August 2020. Seventy-three patients were excluded from the study (lack of smartphone in 37 patients, lack of or poor Internet connectivity in 29 patients, privacy issues in five patients, and loss of interest at follow-up in two patients). The remaining 200 patients were randomly divided into two groups through an online simple randomization generator. Group A included patients who received free-of-charge videoconferencing teleconsultations using a vendor that complies with the Health Insurance Portability and Accountability Act (Zoom application), and every meeting had its number and password (13) to ensure the maintenance of privacy the patient and data encryption, while group B included patients who received regular care as a control group. All patients signed an informed written consent during the hospital stay, and the design of the study was evaluated and approved by the ethical committee of the cardiology department in Ain Shams University following the updated Declaration of Helsinki in 2008. Group A patients who needed the help of a family member through the teleconsultation were identified and consented for approval to receive help during the videoconferencing teleconsultation.

All patients were assessed before discharge to obtain relevant clinical data including age, gender, risk factors for CAD such as hypertension (HTN), diabetes mellitus (DM), smoking status, obesity (body mass index), educational level, socioeconomic status (14), the territory of AMI, procedural success by assessment of post-procedural thrombolysis in MI (TIMI) flow, and procedural or in-hospital complications. Before discharge, all patients underwent transthoracic echocardiography to assess the left ventricular ejection fraction, and medical treatment upon discharge was prescribed according to the European Society of Cardiology 2017 guidelines for the treatment of STEMI (2). A supervised exercise-based phase II cardiac rehabilitation program was also prescribed to all patients upon discharge as per our hospital protocol.

The group A patients were contacted to schedule free-of-charge videoconferencing teleconsultations using the Zoom application starting 1 week after discharge. At least one virtual visit was arranged for every patient per month for the 3-month follow-up period, during which at least one F2F hospital or clinic visit was done (throughout the follow-up period, the frequency of teleconsultation was three visits in 95% of patients, four visits in 3% of patients, and five visits in 2% of patients). All patients were instructed that the teleconsultations are not an alternative to the usual medical care in emergencies and that, if any alarming symptom occurred (such as persistent chest pain), the patients had to seek an emergency medical checkup. Before each virtual visit, the patients were asked to check their BP using an upper-arm automated device through a community pharmacy (a free-of-charge service) or a home-based measurement if available. The subjects were instructed to measure BP in a standardized way: measurements were taken from the dominant arm while sitting and with the feet touching the ground and with the back supported, arm at the level of the heart, and avoiding heavy meals or exercise in the previous 30 min before measurement (15). The BP readings were manually sent by patients as a text message to a dedicated investigator. Every virtual visit lasted about 20 min and included reviewing the symptoms of the patient, checking adherence to medications, up-titration of doses, if required, to reach target doses or a maximum tolerated dose according to the European Society of Cardiology 2017 guidelines for the treatment of STEMI, and, ordering for requested laboratory investigations according to need. An important part of virtual visits was specified to patient education and enforcing the basics of cardiac rehabilitation with encouragement to follow healthy lifestyle measures, especially smoking cessation in smokers, exercise training, and following a healthy diet. The help of a family member had been needed by 11% of patients to attend the videoconferencing teleconsultations.

At 4 months after discharge, the patients were reviewed, and a survey was done to collect data from the patients to assess their satisfaction with the telecardiology services and to compare it with traditional F2F visits as regards personal contact, time of visit, and overall satisfaction using a survey that was developed by RAND Corporation (16). During this period of follow-up, the occurrence of MACE including cardiovascular death, HF hospitalization, revascularization of the target vessel, MI, or cerebrovascular stroke (CVS) was reported (17) in addition to their level of adherence to medications, smoking cessation in smokers, and participation in phase II exercise-based cardiac rehabilitation program in the specialized cardiac rehabilitation clinic in our hospital. The patients were considered to be adherent to medications or cardiac rehabilitation programs when they achieve at least 80% coverage of the mean proportion of days (18). Among smokers, we considered any incidence of smoking within the 3-month follow-up period to be non-adherence.

The group B patients were instructed upon discharge to have their regular medical follow-up, and at least one F2F medical contact was required during the first 3 months after their AMI. They were reviewed also 4 months after discharge, and reporting of MACE, adherence to medications, smoking cessation among smokers, and participation in cardiac rehabilitation was done (17). Table 1 shows the study flow chart.

**Table 1:**
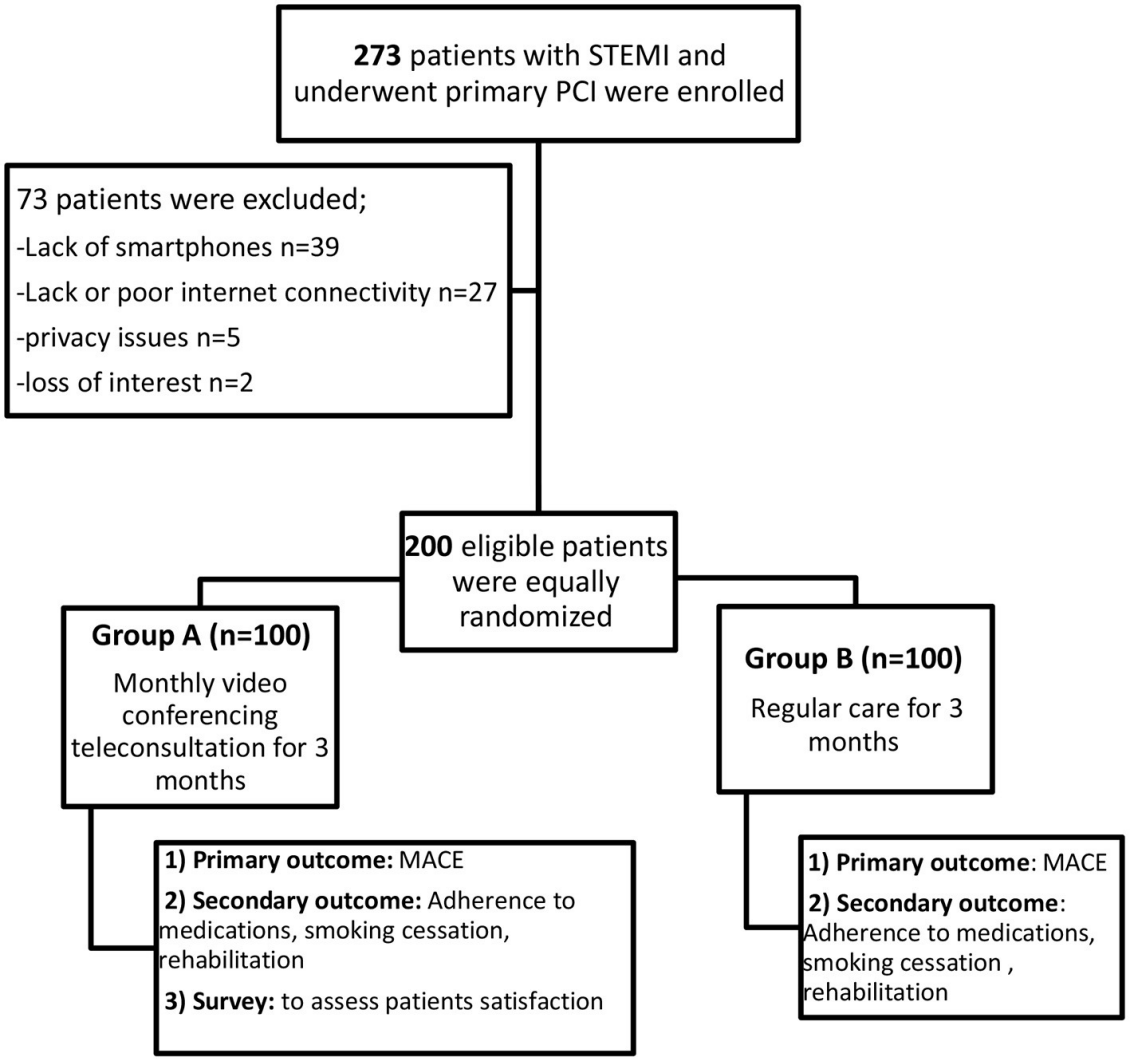
Flow chart of the study. MACE, major adverse cardiac events; PCI, percutaneous coronary intervention; STEMI, ST-segment elevation myocardial infarction.

### Statistical Analysis

Data was collected, coded, and entered into the Statistical Package for Social Science (IBM SPSS), version 20. Qualitative data was presented as frequency and percentage. The *t*-test was used in comparing parametric variables, while chi-square test was used for contingency tables.

## Results

### Baseline Characteristics

Eligible patients were randomized into 100 patients representing the study group (group A) in addition to 100 patients representing the control group (group B). There was no significant difference between both groups as regards age and gender. There was also no significant difference between them on comparing the risk factors for CAD (smoking, HTN, and DM) and previous history of ischemic heart disease such as chronic stable angina, MI, PCI, or CABG. Both groups shared nearly similar socioeconomic status and educational level. This data is shown in Table 2.

**Table 2:** Comparison of baseline characteristics, MACE, adherence to medications, and lifestyle measures between both groups.

	**Group A (*n* = 100)**	**Group B(*n* = 100)**	***p*-value**
**Baseline characteristics**			
Age in years (mean ± SD)	56.2 ± 9.3	55.8 ± 11.2	0.8
Male gender, %	73	70	0.64
HTN, %	35	37	0.77
DM, %	36	35	0.88
Smoking, %	72	67	0.44
IHD, %	24	26	0.74
**Socioeconomic class**			
Very low, %	26	24	0.92
Low, %	39	41	
Middle, %	34	33	
High, %	1	2	
**MACE**			
Overall MACE, %	11	14	0.52
Cardiovascular mortality, %	1	2	0.56
CVS, %	1	1	1
MI, %	4	6	0.5
TVR, %	3	6	0.3
HF hospitalization, %	5	9	0.27
**Medication, smoking cessation and rehabilitation adherence**			
Aspirin, *n* (%)	96 (96.9%)	93 (94.9%)	0.64
P2Y12 inhibitor, *n* (%)	96 (96.9%)	93 (93%)	0.64
Statin, *n* (%)	86 (86.8%)	70 (71.4%)	0.02^*^
BB, *n* (%)	86 (86.8%)	80 (81.6%)	0.51
ACE/ARB, *n* (%)	62 (83.7%)	42 (60%)	0.006^*^
Rehabilitation, *n* (%)	62 (62.6)	29 (29.6)	≤ 0.001^*^
Smoking cessation, *n* (%)	54 (75%)	26 (38.8%)	≤ 0.001^*^

*HTN, hypertension; DM, diabetes mellitus; IHD, ischemic heart disease; CVS, cerebrovascular stroke; TVR, target vessel revascularization; HF, heart failure; MACE, major adverse cardiac events; ACEI, angiotensin-converting enzyme inhibitors; ARB, angiotensin II receptor blockers; BB, B-blockers.*

* *p-value is significant if < 0.05*.

### Major Adverse Cardiovascular Events

The primary outcome of this study was the incidence of MACE during the first 4 months after discharge that did not differ significantly between both groups (MACE in group A was 11 vs. 14% in group B, *p* = 0.521). There was also no significant difference in comparing the composites of MACE in this study (cardiovascular mortality, MI, HF hospitalization, CVS, and target vessel revascularization) as shown in Table 2.

### Adherence of Patients to Medications and Cardiac Rehabilitation

By the end of the 4-month follow-up period, one case of cardiovascular mortality was recorded in group A, while two cases were recorded in group B. The secondary outcome of this study was to compare the adherence of patients to medications and cardiac rehabilitation between both groups. Adherence to medications and cardiac rehabilitation were compared in the remaining population between both groups at the end of the 4-month follow-up period. Both groups showed a comparable high incidence of good adherence to aspirin and P2Y12 inhibitor (96% good adherence in group A vs. 93% in group B, *p* = 0.64). They also showed a comparable incidence of good adherence to beta-blockers (BB) (86 vs. 80%, *p* = 0.5). However, the group A patients showed a higher incidence of good adherence to statins (86 vs. 70%, *p* = 0.024), angiotensin-converting enzyme inhibitors/angiotensin II receptor blockers (ACEI/ARB) among patients taking ACEI/ARB (83.7 vs. 60%, *p* = 0.006), smoking cessation among smokers (75 vs. 38.8%, *p* = 0.001), and cardiac rehabilitation (62 vs. 29%, *p* = 0.001) as shown in Table 2.

### Patient Satisfaction Survey

**Figure 1:**
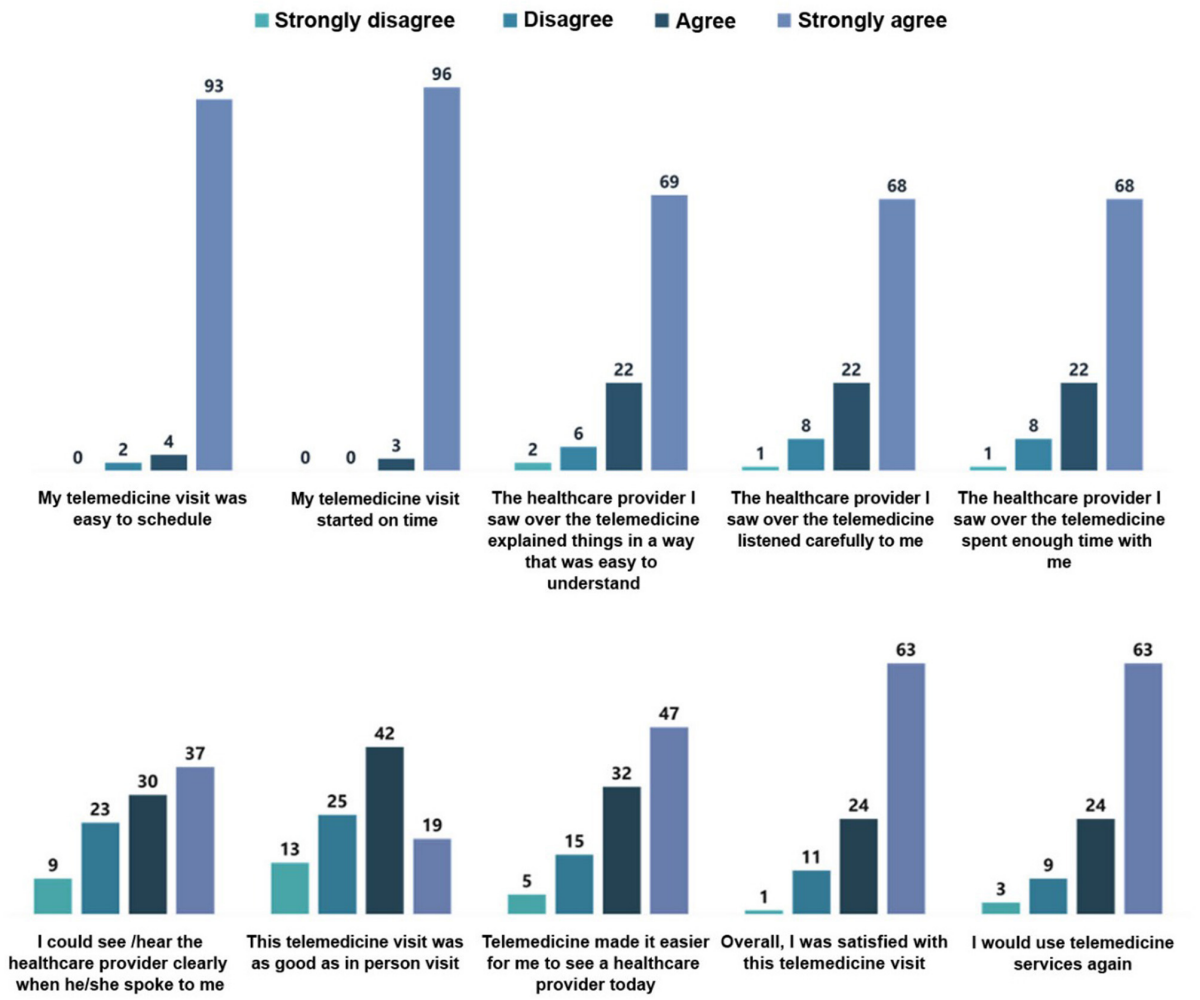
Patient satisfaction survey.

A survey was done aiming to assess the overall satisfaction of group A with telemedicine service. Only 11% of group A reported that they previously used telemedicine, while 34% of patients needed help from a family member to initiate and complete the telemedicine exam. Sixty-one percent of patients stated that the telemedicine visits were as good as the clinic visits, while 87% of patients were satisfied with telemedicine. Data derived from this survey is illustrated in Figure 1.

## Discussion

Lost follow-up after the discharge of patients with AMI is associated with sub-optimal or inappropriate medication dosing and up-titration of BBs and ACEI or ARB, if required, in addition to decreased adherence to these medications and secondary preventive lifestyle measures, while increasing adherence following MI to secondary preventive measures including guideline-recommended medical treatment and healthy lifestyle measures is associated with improved cardiovascular outcome (19). Non-adherence is not simply related to the non-compliance of patients only, including forgetfulness or reluctance, but is also related to other various factors, including economic, drug-related, and healthcare system-related factors (20), and it is associated with increased mortality and morbidity (3). Furthermore, it was found that adherence declines progressively as time passed from the acute event (21).

In this study, we aimed to encourage adherence to guideline-recommended medical treatment and lifestyle measures early after discharge when adherence is expected to be at the maximum to maintain a high level of adherence in those who are adherent and to increase the level of adherence in those who are not adherent. Patients who received videoconferencing teleconsultations in addition to their usual medical care received an explanation of the value of secondary preventive measures and side effects of the prescribed drugs in addition to up-titration of BB and ACEI or ARB doses, if needed, to face barriers against medication adherence. We also asked the patients to measure their BP through an automated upper-arm device before each teleconsultation to encourage them to participate actively in their BP management (15).

The primary outcome of this study was to compare MACE between both groups that were slightly lower in group A (11 vs. 14%) despite a significantly better adherence to statin therapy, ACEI or ARB, smoking cessation, and cardiac rehabilitation. This may be related to the short-term follow-up period in our study and the non-significant difference in adherence to dual antiplatelet therapy and BBs. Many studies in literature concluded that adherence to guideline-recommended medical treatment after MI is associated with a long-term better outcome, reduced MACE, and lower mortality (22). Despite various definitions and incidence of MACE after STEMI, MACE is still considered to be the major cause of morbidity and mortality in these patients. MACE is commonly used in cardiovascular research to assess the effectiveness and safety of different treatment strategies that target MACE reduction after STEMI to reduce morbidity and mortality.

While the secondary outcome was to compare medication, smoking cessation, and rehabilitation adherence between both groups, group A showed a non-significantly higher incidence of adherence to aspirin and P2Y12 inhibitors (96 and 96% vs. 93 and 93%), and this may be explained by the selective focusing upon discharge of patients with STEMI presented to our institute to maintain adequate adherence to dual antiplatelet therapy to reduce the incidence of stent thrombosis as a significant percentage of patients are not included under the umbrella of health insurance. A study of anti-platelet adherence following MI showed an early high adherence 1 month after the acute event (90.3%) that declined to (46.3%) after only 1 year (23). Another study showed that 8% of patients discharged after acute coronary syndrome (ACS) discontinued aspirin therapy (24). Non-adherence to dual anti-platelet therapy following MI and coronary intervention is associated with an increased risk of stent thrombosis and spontaneous MI (25). Telemedicine services showed better adherence to aspirin in some studies. Short messages sent to patients daily for 1 month after intervention for ACS decreased aspirin non-adherence from 6.4 to 3.6% (26). Four telephone calls within a year following coronary stenting also improved adherence to aspirin and clopidogrel (27).

Group A also showed a non-significantly higher incidence of adherence to BBs in patients on BB therapy (86 vs. 80%). A study showed that patients discharged on BBs had good adherence at 1 month (85%) that declined to 63% at 6 months and 61% at 1 year, and that adherence was lower in patients who did not start BB therapy during hospital stay (28). Another study showed that 12% of patients discontinue BBs at 6 months (24).

On the other hand, group A showed a significantly higher incidence of adherence to ACEI or ARB (83.7 vs. 60%) and statin therapy (86 vs. 70%) in comparison to group B. A study showed that 20% of patients discontinued ACEI at 6 months, while 13% of patients discontinued statin therapy at 6 months (24), and another study showed that MACE following myocardial infarction was significantly lower after 2 years in patients who achieved adequate adherence to ACEI and statins in comparison to those who were partially adherent or poorly adherent (18.9 vs. 24.7 vs. 26.3%) (29). A study showed that voice messaging for 12 months after ACS improved adherence to BBs, statins, ACEI or ARB, and clopidogrel from 73.9 to 89.3% (18).

Group A also showed a significantly higher incidence of adherence to smoking cessation among smokers (75 vs. 38.8%) and participation in supervised exercise-based cardiac rehabilitation (62 vs. 29%). Although the routine referral of smokers to smoking cessation clinics is not adopted in our institute, all patients are encouraged before discharge to attach to a cardiac rehabilitation program that includes lifestyle modification advice in addition to exercise-based rehabilitation. A study of the effect of smoking cessation on mortality after MI concluded that the number needed to quit smoking to save a single life is 13 (30), and another study showed that more successful maintenance of smoking cessation was related to longer duration of hospital stay that was attributed to the longer cessation of smoking during admission and more time to advise patients as regards the importance of quitting smoking (31). It was advised that all smokers should be offered in-hospital counseling with close follow-up after discharge while supporting the long-term success of smoking cessation using medications to relieve withdrawal symptoms (32). Many studies also evaluated cardiac rehabilitation after MI and concluded that it has beneficial effects in reducing the risk factors of CAD, morbidity, and mortality in addition to improving the quality of life (33, 34). Moreover, adherence to lifestyle modification is significantly associated with medication adherence in post-AMI patients (35). These findings may give a chance to digital healthcare services that could provide an improvement in the adherence to the guideline recommendations following AMI.

In this study, videoconferencing was the tool to communicate with patients as it enabled the eye-to-eye contact that is missing in other forms of telemedicine to enhance the doctor–patient relationship. Collected data from a meta-analysis in 2020 stated that videoconferencing teleconsultations were effective in the assessment and improvement of the health status of patients with a high degree of patient satisfaction (36). However, a review in 2019 reported that videoconferencing is not more than equivalent to F2F consultations (37). Videoconferencing was used in the remote care of cardiac patients in different fields of cardiovascular medicine that was comparable to F2F visits (38–40).

The use of videoconferencing consultations increased with the COVID-19 pandemic (41), and it appeared to be beneficial in keeping social distancing, avoiding the spread of infection, and reducing unnecessary visits, with better acceptance from patients and healthcare workers in comparison to phone call telemedicine services (42). The increasing availability of smartphones among the population allowed their use in the current study for doctors to connect with patients even with low to middle socioeconomic status compared to laptops. Virtual visit hosting took place using a free-of-charge application (Zoom). Its choice was based on the balance it carries between maintaining privacy and being without difficulty for patients to use. Although there are more secure options like the Microsoft Teams, Zoom application has the advantage of being an easy one to use and still complies with the Health Insurance Portability and Accountability Act (43, 44). The lockdown for the COVID-19 pandemic increased the familiarity of the population with this application as it is widely used in scientific, academic, and work-related meetings, which reduced the failure to accomplish virtual visits from the first trial. Enhancement of telemedicine service delivery was done through the help of a family member to those who did not have a smartphone or could not use the technology. Patients who were not familiar with the application were also given a trial to test their audiovisual connection before the start of their visits. However, rescheduling of the first virtual visit was done in 8% of patients due to difficulty in dealing with the application and in 11% of patients due to poor or interrupted Internet connectivity. Later visits were only rescheduled due to Internet connection issues. Another obstacle in the scheduling of visits occurred in patients who were dependent on a family member as it was more difficult to arrange. Despite these technical issues, Zoom application remains one of the most widely used applications in the field of telemedicine due to many factors (freely available, secure, and easy to use) (44, 45). This was confirmed by the high degree of patient satisfaction with the ease of scheduling and use of this application.

### Patient Satisfaction With Telemedicine Service

In this study, patients were satisfied with these virtual visits after discharge. A large percentage of patients were dependent on a family member who is capable of dealing with video calls. This relatively high level of satisfaction may be attributed to ease of contact with a specialist early after discharge and to the saved time of travel and money, in addition to introducing detailed data regarding prescribed medications and lifestyle measure value and side effects. However, missing real contact with healthcare providers was considered a barrier for some patients to accept telemedicine services, especially when presented as an alternative to clinic or hospital visits. Many studies showed a high level of patient satisfaction with telemedicine services (46, 47).

### Limitations of the Study

This study took place in a single center (tertiary University hospital) that nearly excluded unintentionally patients with high socioeconomic status; thus, not all cultural and educational categories were represented. The current legal issues also allowed this study to introduce telemedicine services to the study group as an additional free-of-charge service, not as an alternative to F2F visits, so that they receive additional care without added financial burden. However, the cost-effectiveness of this service could not be analyzed as both groups received the usual regular care. It is well-known that maintained adherence to medications and lifestyle measures is strongly related to better outcomes in the long term, so the actual analysis of cost-effectiveness needs a further study that may introduce videoconferencing teleconsultations as an alternative to some face-to-face visits in a more prolonged duration of follow-up.

Additionally, lack of or poor Internet connectivity and lack of technological support (24% of initially enrolled patients) remain as significant challenges facing the wide application of telemedicine services. Finally, this study only addressed short-term outcome that did not differ in the primary outcome (MACE), although group A showed better adherence to medications and lifestyle measure that has a better outcome as regards MACE on the long-term follow-up.

## Conclusions

Telemedicine may provide additional benefit to short-term regular care after primary PCI to STEMI patients through videoconferencing teleconsultations using smartphones by increasing their adherence to the guideline-recommended medications and lifestyle measures (smoking cessation and cardiac rehabilitation) without a significant difference in the short-term MACE. These videoconferencing teleconsultations gained a high level of satisfaction from patients. However, lack of technology and poor Internet connectivity are still important challenges that prevent a significant portion of the population from receiving telemedicine services.

## Data Availability Statement

The original contributions generated for this study are included in the article/supplementary material, further inquiries can be directed to the corresponding author/s.

## Ethics Statement

The studies involving human participants were reviewed and approved by Ethics committee of Cardiology department, faculty of medicine, Ain Shams University. The patients/participants provided their written informed consent to participate in this study.

## Author Contributions

HK contributed to the idea and conception, data collection, data analysis, and writing the draft of the manuscript. MH contributed to the study design, data collection, data analysis, and writing the manuscript. IB contributed to the study design, data collection, data analysis, statistical analysis, and writing the final manuscript. All authors contributed to the article and approved the submitted version.

## Conflict of Interest

The authors declare that the research was conducted in the absence of any commercial or financial relationships that could be construed as a potential conflict of interest.
